# Development and validation of a visualized prediction model for early miscarriage risk in patients undergoing IVF/ICSI procedures: a real-world multi-center study

**DOI:** 10.3389/fendo.2023.1280145

**Published:** 2024-02-14

**Authors:** Meng Zhang, Xiaohui Ji, Xinye Hu, Yingying Zhu, Haozhe Ma, Hua Xu, Xiaolin La, Qingxue Zhang

**Affiliations:** ^1^ Reproductive Medicine Center, Sun Yat-sen Memorial Hospital, Sun Yat-sen University, Guangzhou, China; ^2^ Guangdong Provincial Clinical Research Center for Obstetrical and Gynecological Diseases, Guangzhou, China; ^3^ Department of Obstetrics and Gynaecology, People's Hospital of Changji Hui Autonomous Prefecture, Changji, China; ^4^ Medical College, Jinan University, Guangzhou, China; ^5^ Division of Clinical Research Design, Sun Yat-sen Memorial Hospital, Sun Yat-sen University, Urumqi, Xinjiang, China; ^6^ Center of Reproductive Medicine, The First Affiliated Hospital of Xinjiang Medical University, Urumqi, China

**Keywords:** early miscarriage, nomogram, IVF-ET, individualized prediction, pregnancy outcome

## Abstract

**Background:**

This study focuses on the risk of early miscarriage in patients undergoing *in vitro* fertilization (IVF) or intracytoplasmic sperm injection (ICSI). These patients commonly experience heightened stress levels and may discontinue treatment due to emotional burdens associated with repeated failures. Despite the identification of numerous potential factors contributing to early miscarriage, there exists a research gap in integrating these factors into predictive models specifically for IVF/ICSI patients. The objective of this study is to develop a user-friendly nomogram that incorporates relevant risk factors to predict early miscarriage in IVF/ICSI patients. Through internal and external validation, the nomogram facilitates early identification of high-risk patients, supporting clinicians in making informed decisions.

**Methods:**

A retrospective analysis was conducted on 20,322 first cycles out of 31,307 for IVF/ICSI treatment at Sun Yat-sen Memorial Hospital between January 2011 and December 2020. After excluding ineligible cycles, 6,724 first fresh cycles were included and randomly divided into a training dataset (n = 4,516) and an internal validation dataset (n = 2,208). An external dataset (n = 1,179) from another hospital was used for validation. Logistic and LASSO regression models identified risk factors, and a multivariable logistic regression constructed the nomogram. Model performance was evaluated using AUC, calibration curves, and decision curve analysis (DCA).

**Results:**

Significant risk factors for early miscarriage were identified, including female age, BMI, number of spontaneous abortions, number of induced abortions and medical abortions, basal FSH levels, endometrial thickness on hCG day, and number of good quality embryos. The predictive nomogram demonstrated good fit and discriminatory power, with AUC values of 0.660, 0.640, and 0.615 for the training, internal validation, and external validation datasets, respectively. Calibration curves showed good consistency with actual outcomes, and DCA confirmed the clinical usefulness. Subgroup analysis revealed variations; for the elder subgroup (age ≥35 years), female age, basal FSH levels, and number of available embryos were significant risk factors, while for the younger subgroup (age <35 years), female age, BMI, number of spontaneous abortions, and number of good quality embryos were significant.

**Conclusions:**

Our study provides valuable insights into the impact factors of early miscarriage in both the general study population and specific age subgroups, offering practical recommendations for clinical practitioners. We have taken into account the significance of population differences and regional variations, ensuring the adaptability and relevance of our model across diverse populations. The user-friendly visualization of results and subgroup analysis further enhance the applicability and value of our research. These findings have significant implications for informed decision-making, allowing for individualized treatment strategies and the optimization of outcomes in IVF/ICSI patients.

## Introduction

Early miscarriage can result in complications such as bleeding and infection, and can also have a negative impact on the mental health of women, causing feelings of depression, loss, and self-blame. These issues can significantly affect the daily lives and interpersonal relationships of women. Additionally, early miscarriage can damage the endometrium and impair women’s fertility. Early miscarriage is a prevalent occurrence during the initial trimester, affecting approximately 15% to 20% of pregnancies that have been confirmed (1–3). Moreover, it is estimated that up to 80% of all miscarriages occur during the first trimester of pregnancy (4, 5). The risk of miscarriage significantly decreases after 12 weeks of gestation, with studies suggesting that the incidence of miscarriage drops sharply to approximately 1% after this point (6). Therefore, this article will focus specifically on the risk of first trimester miscarriage. Women who have undergone assisted reproductive technology (ART) to conceive have been found to experience higher levels of stress than those who conceive naturally (7).. This has led some patients to discontinue further treatment due to the emotional burden associated with repeated IVF failures (8, 9). Investigating the factors that contribute to early miscarriage in IVF pregnancies can provide valuable insights into the occurrence of IVF-related miscarriages. By identifying and studying these factors, appropriate preventive measures can be taken to decrease the incidence of IVF-related miscarriages, thereby safeguarding the physical and mental well-being of women.

Although numerous potential factors have been identified as contributors to early miscarriages, there is a lack of research that integrates these factors into predictive models for IVF/ICSI patients. Therefore, our study aimed to develop a user-friendly nomogram that visually predicts the probability of first-trimester miscarriage for IVF/ICSI patients. The nomogram was validated internally and externally, and we hope that it can be used by clinicians to identify high-risk patients early and make informed medical decisions. Additionally, the nomogram can help patients understand the factors that contribute to early miscarriage and how to decrease their risk, thereby reducing psychological stress.

## Methods

### Participants

This retrospective study finally included a total of 6,724 pregnancies achieved through first fresh embryo transfer cycles between January 2011 and December 2020 at the Reproductive Medicine Center of Sun Yat-Sen Memorial Hospital in Guangzhou, China. Serum β-human chorionic gonadotropin (β-hCG) levels were measured 14 days after cleavage embryo transfer or 12 days after blastocyst transfer to confirm clinical pregnancy, defined by a serum β-hCG level of ≥25 IU/L. Early miscarriage was defined as miscarriage occurring before 12 weeks’ gestation. The study group was divided into upper (≥35 years) and lower age (<35 years) groups based on the age of 35. The study was approved by the Institutional Review Boards of Sun Yat-Sen Memorial Hospital (SYSEC-KY-KS-2021-121) and the First Affiliated Hospital of Xinjiang Medical University (K202106-17) and is reported in accordance with the TRIPOD statement (10).

### Data collection

**Figure 1:**
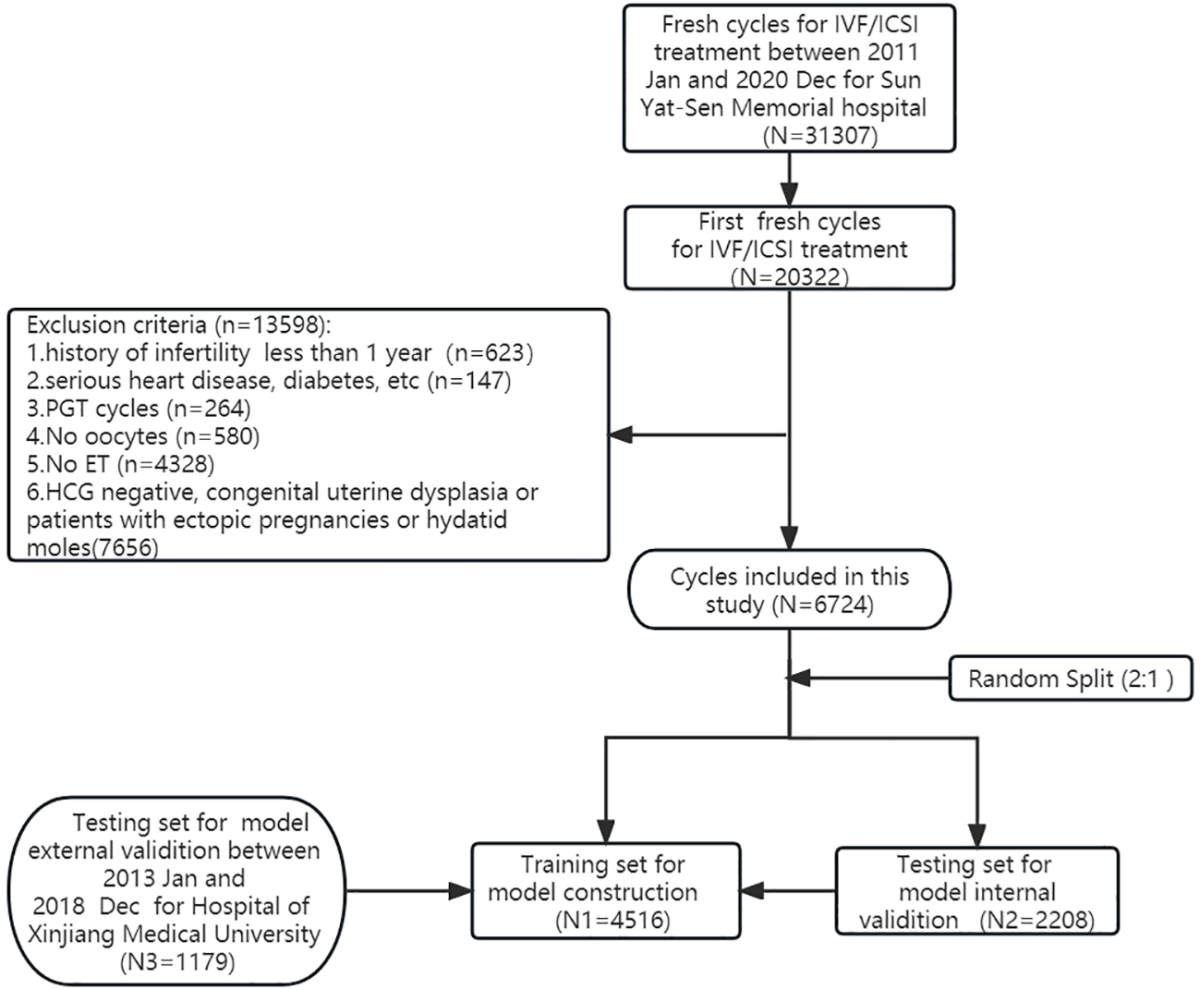
General flowchart of modeling algorithm.

**Figure 1** illustrates the selection process of cycles for this retrospective study conducted at Sun Yat-Sen Memorial Hospital between January 2011 and December 2020. Out of a total of 31,307 fresh cycles, we identified and included 20,322 first fresh cycles for IVF/ICSI treatment. After excluding specific cases, a total of 6,724 cycles were analyzed in this study. All patients’ cycles (6,724 cycles) were randomly divided into a training dataset (4,516 cycles) and an internal validation dataset (2,208 cycles) at a ratio of 2:1 using random number sampling techniques. Additionally, we collected 1,179 cycles as external validation data from the First Affiliated Hospital of Xinjiang Medical University between January 2013 and December 2018. The training dataset was used to screen variables and construct a predictive nomogram model, while both the internal and external validation datasets were utilized to evaluate the model’s performance. In our research, the external dataset, training dataset, and internal validation dataset all utilized internationally recognized diagnostic criteria for infertility (11). Furthermore, the inclusion and exclusion criteria for the study subjects were consistent across these datasets. We also employed a uniform methodology during the data collection and analysis processes, guaranteeing that our assessment of different regional populations is conducted on a comparable basis. The characteristics of patients in the training dataset, internal validation dataset, and external dataset are detailed in **Table 1**.

**Table 1:** Comparison of characteristics and medical history of the study population between training dataset and validation dataset.

Variable	Training dataset N1 = 4516	Internal validation dataset N2 = 2208	External validation dataset N3 = 1179	*P* value
Female age (years)	31.42 ± 4.22	31.58 ± 4.26	31.97 ± 3.98^a,b^	<0.001
Male age (years)	33.88 ± 5.28	33.92 ± 5.13	–	–
Female BMI (kg/m^2^)	21.44 ± 2.86	21.37 ± 2.73	22.93 ± 3.27^a,b^	<0.001
Duration of infertility (years)	4.23 ± 2.89	4.31 ± 2.99	4.22 ± 2.86	0.490
Type of Infertility				0.010
Primary	2213 (49%)	1050 (47.55%)	624 (52.97%)^a,b^	
Secondary	2303 (51%)	1158 (52.45%)	554 (47.03%)	
Insemination method				–
IVF	3237 (71.68%)	1579 (71.51%)	–	
ICSI	1279 (28.32%)	629 (28.49%)	–	
Endometriosis				0.047
No	4346 (96.24%)	2139 (96.88%)	1150 (97.62%)	
Yes	170 (3.76%)	69 (3.13%)	28 (2.38%)^a^	
Tubal factor				<.001
No	2544 (56.33%)	1220 (55.25%)	369 (31.32%)	
Yes	1972 (43.67%)	988 (44.75%)	809 (68.68%)^a,b^	
Ovulation disorder				0.902
No	4308 (95.39%)	2103 (95.24%)	1126 (95.59%)	
Yes	208 (4.61%)	105 (4.76%)	52 (4.41%)	
Male factor				0.468
No	3789 (83.9%)	1870 (84.69%)	979 (83.11%)	
Yes	727 (16.1%)	338 (15.31%)	199 (16.89%)	
Smoking				–
No	4496 (99.56%)	2196 (99.46%)	–	
Yes	20 (0.44%)	12 (0.54%)	–	
Dysmenorrhea				0.009
No	2993 (66.28%)	1433 (64.9%)	712 (61.49%)	
Yes	1523 (33.72%)	775 (35.1%)	446 (38.51%)^a,b^	
Number of spontaneous abortions	0.16 ± 0.48	0.17 ± 0.48	0.42 ± 0.77^a,b^	<0.001
Number of induced abortions and medical abortions	0.30 ± 0.64	0.32 ± 0.66	–	<0.001
AFC	17.15 ± 8.66	17.28 ± 8.62	11.22 ± 7.32^a,b^	<0.001
Basal FSH (IU/L)	7.97 ± 2.73	7.92 ± 2.47	7.93 ± 3.44	0.800
Basal LH (IU/L)	5.22 ± 3.43	5.28 ± 3.44	5.98 ± 27.08^a,b^	0.055
Basal E2 (ng/ml)	49.48 ± 70.2	49.75 ± 73.0	53.63 ± 160.31	0.363
Basal T (ng/ml)	1.56 ± 3.24	1.58 ± 3.46	0.68 ± 3.67^a,b^	<0.001
Gn dose (IU)	2104.14± 803	2112.64± 827	2700.83 ± 897.56^a,b^	<0.001
Gn days	11.46 ± 2.72	11.51 ± 2.79	12.13 ± 2.41^a,b^	<0.001
FSH (IU/L) on hCG day	17.71 ± 7.51	25.35± 322	–	–
E2 (ng/ml) on hCG day	2845.17 ± 1265	2865.86 ± 1255	2106.14 ± 1656^a,b^	<0.001
LH (IU/L) on hCG day	2.32 ± 3.79	2.30 ± 2.29	1.45 ± 1.56^a,b^	<0.001
P (IU/L) on hCG day	1.06 ± 0.57	1.06 ± 0.51	1.96 ± 1.41^a,b^	<0.001
Number of oocytes on hCG day	2.88 ± 2.00	2.87 ± 1.96	–	–
Endometrial thickness (mm)	11.91 ± 2.61	11.82 ± 2.53	10.87 ± 2.15^a,b^	–
Retrieved oocytes (n)	11.40 ± 5.11	11.45 ± 5.12	10.67 ± 5.26^a,b^	<0.001
2PN	7.19 ± 3.65	7.16 ± 3.62	6.41 ± 3.59^a,b^	<0.001
MII oocytes	9.78 ± 4.53	9.83 ± 4.57	5.92 ± 511^a,b^	–
Number of available embryos	5.49 ± 3.23	5.46 ± 3.22	5.39 ± 5.04	0.679
Number of good quality embryos	2.48 ± 2.23	2.43 ± 2.18	2.70 ± 2052^a,b^	0.004
Number of embryos transferred	1.34 ± 0.99	1.37 ± 1.00	1.85 ± 0.41^a,b^	<0.001
Number of GQB per transferred	1.30 ± 0.75	1.30 ± 0.77	1.37 ± 0.36^a,b^	0.038
Early miscarriage				
No	4090 (90.57%)	1975 (89.45%)	1019 (86.5%)	0.0002
Yes	426 (9.43%)	233 (10.55%)	159 (13.5%)	

AFC, Antral follicle count; FSH, Follicle-stimulating hormone; LH, Luteinizing hormone; Basal T, basal testosterone; Gn, gonadotropin; HCG, human chorionic gonadotropins; P, progesterone; E2, estradiol; MII, metaphase II; 2PN, fertilized oocytes GQB, Good quality blastocyst.

a: *P*< 0.05 between External validation dataset and Training dataset.

b: *P*< 0.05 between External validation dataset and Internal validation dataset.

There were no significant differences between Training dataset and Internal validation dataset.

### Statistical analysis

We performed statistical analysis using R software (version 4.2.1, http://www.r-project.org/). Participant characteristics were summarized using mean and standard deviation for continuous variables and frequency and percentage for categorical variables. We used t-tests to compare differences between continuous variables and the χ^2^ test or Fisher’s exact test for categorical variables. In the training dataset, we used LASSO regression to identify potential risk variables with non-zero coefficients, and univariable logistic regression to identify significant variables. We then conducted multivariable logistic regression (MLR) to identify significant prognostic factors associated with early miscarriage. All significant variables either from LASSO regression or univariable logistic regression were selected in MLR model. Then the significant factors identified in MLR were used to construct predictive models, and a nomogram was graphically visualized using the R package “rms”. Moreover, we performed subgroup analyses based on age (≥35 years or <35 years) and constructed corresponding subgroup nomograms.

Model performance was assessed based on three dimensions: discrimination, calibration, and clinical usefulness. Discrimination was measured using the area under the curve (AUC) of the receiver operating characteristic (ROC) curve. Calibration was evaluated using calibration curves and unreliability tests. The clinical utility of the nomogram was assessed using decision curve analysis (DCA) by quantifying the standardized net benefit at different threshold probabilities. During the evaluation phase, we utilized three R packages (“pROC”, “Resource Selection”, “rmda”) for analysis. All reported statistical significance levels were two-sided, and statistical significance was set at 0.05.

## Results

### Characteristics of patients

**Table 1** shown the comparation of baseline and cycle characteristics between training set, internal validation set, and external validation set. Training set and internal validation set were comparable. However, statistical differences were observed between the external validation set and the training set/internal validation set for most variables. Specifically, patients in the external validation dataset exhibited higher age, BMI, and number of good quality embryos, lower antral follicle count (AFC), basal testosterone (T), and luteinizing hormone (LH) levels on hCG day, as well as a lower number of retrieved oocytes, 2PN and MII oocytes, Additionally, they required higher doses of gonadotropin (Gn),Gn days, and exhibited higher levels of estradiol (E2) and progesterone (P) on hCG day. Furthermore, the external validation set had a higher primary infertility rate and a higher prevalence of tubal factor infertility and dysmenorrhea. The early miscarriage were 9.43%, 10.55% and 13.50% in these three datasets, respectively, and the difference were statistical (*P*=0.0002).

We then divided total population into elder (age ≥35 years) and younger subgroups (age <35 years). The comparison of characteristics and medical history of the study population between training dataset and validation dataset were shown in **Supplementary Table 1** for elder women and **Supplementary Table 2** for younger women. Elder women had higher duration of infertility (years) of 5.28 than 3.91 for younger women and 4.23 for total women. Younger women had higher primary infertility rate of 55.17%, than 28.52% for elder women and 49% for total women. Besides, younger women had higher male factor rate(16.97%) and dysmenorrhea rate(36.07%) than elder women (13.21% and 25.93%). Elder women had higher number of spontaneous abortions and number of induced abortions and medical abortions than younger women. However, elder women had lower AFC, retrieved oocytes(n),2PN (8.71, 13.76, 10.06, 6.36) than younger women (10.56, 18.17, 11.8, 7.44). Elder women had higher early miscarriage of 17.32% than 7.06% for younger women.

### Variable screening and construction of nomogram prediction model

The comparison of characteristics between miscarriage group and unmiscarriage group in the train dataset were summarized in **Supplementary Table 3**. Univariable logistic regression demonstrated that female age, male age, female body mass index (BMI), type of infertility, tubal factor, the number of spontaneous abortions, the number of induced abortions, AFC, basal FSH (IU/L), Gn days, FSH (IU/L) on hCG day, E2 (ng/ml) on hCG day, retrieved oocytes (n), MII oocytes, 2PN, number of available embryos, and number of good quality embryos per transferred may potential prognostic factors for miscarriage (**Supplementary Table 3**). Besides, LASSO regression demonstrated female age, female BMI, number of spontaneous abortions and female age (years), type of infertility, male age (years), female BMI(kg/m2), Gn days, MII oocytes, number of good quality embryos, number of available embryos, LH (IU/L) on hCG day, tubal factor, number of spontaneous abortions, number of induced abortions were potential independent risk factors for miscarriage (**Supplementary Figure 1A**), when λ was set at the minimum criteria lambda.1se (the left line) (**Supplementary Figure 1B**).

**Figure 2:**
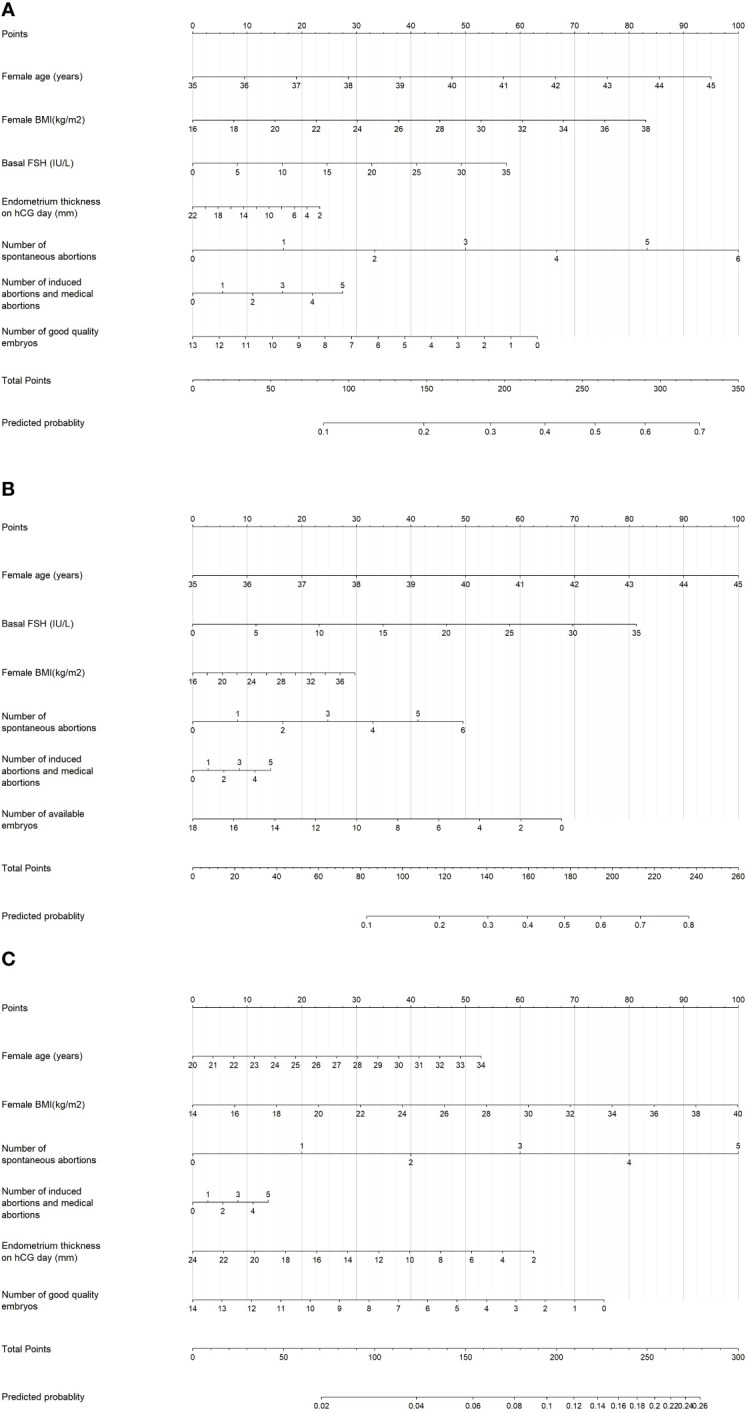
Nomogram for predicting the probability of early miscarriage following IVF/ICSI treatment was developed and validated in three patient groups: **(A)** Total patients, **(B)** Patients aged ≥35 years, and **(C)** Patients aged <35 years.

These significant factors selected by LASSO regression or univariable logistic regression were subsequently included in multivariate logistic model and the results were shown in **Table 2**. It revealed that female age (OR=1.13; 95% CI=1.01-1.15; *P* < 0.001), female BMI (OR=1.05, 95% CI=1.01-1.09, *P*=0.008), number of spontaneous abortions (OR=1.24, 95% CI=1.04-1.47, *P*=0.015), number of induced abortions (OR=1.08, 95% CI=0.93-1.24, *P*=0.325), basal FSH (OR=1.02, 95% CI=0.99-1.06, *P*=0.214), endometrium thickness on hCG day (OR=0.98, 95% CI=0.94-1.02, *P*=0.345) and number of Good quality embryos (OR=0.94, 95% CI=0.90-0.99, *P*=0.021) were independent prognostic factors for early miscarriage for total patients. These seven independent risk factors were incorporated to build predictive model, and then the model was visualized using a nomogram (**Figure 2**). The prediction score of miscarriage can calculated by the nomogram.

**Table 2:** Multivariable logistic regression model of potential prognostic factors.

Variables	β	OR(95%CI)	*P* value
Model 1:Total patients			
Intercept	-7.03		<.0001
Female age (years)	0.12	1.13(1.01-1.15)	<.0001
Female BMI(kg/m2)	0.05	1.05(1.01-1.09)	0.008
Basal FSH (IU/L)	0.02	1.02(0.99-1.06)	0.214
Endometrium thickness on hCG day (mm)	-0.02	0.98(0.94-1.02)	0.345
Number of spontaneous abortions	0.21	1.24(1.04-1.47)	0.015
Number of induced abortions and medical abortions	0.07	1.08(0.93-1.24)	0.325
Number of good quality embryos	-0.06	0.94(0.90-0.99)	0.021
Model 2: Female age ≥35 years			
Intercept	-11.153		<.0001
Female age (years)	0.231	1.26(1.17-1.35)	<.0001
Basal FSH (IU/L)	0.056	1.06(1.00-1.11)	0.036
Female BMI(kg/m2)	0.031	1.03(0.97-1.10)	0.314
Number of spontaneous abortions	0.198	1.22(0.96-1.54)	0.101
Number of induced abortions and medical abortions	0.073	1.08(0.88-1.31)	0.472
Number of available embryos	-0.087	0.92(0.86-0.98)	0.015
Model 3: Female age <35 years			
Intercept	-4.601		<.0001
Female age (years)	0.051	1.05(1.00-1.10)	0.035
Female BMI(kg/m2)	0.053	1.05(1.01-1.10)	0.017
Number of spontaneous abortions	0.274	1.31(1.02-1.70)	0.038
Number of induced abortions and medical abortions	0.039	1.04(0.84-1.30)	0.725
Endometrium thickness on hCG day (mm)	-0.044	0.96(0.91-1.01)	0.102
Number of good quality embryos	-0.073	0.93(0.87-0.99)	0.023

### The validation and performance of nomogram

**Figure 3:**
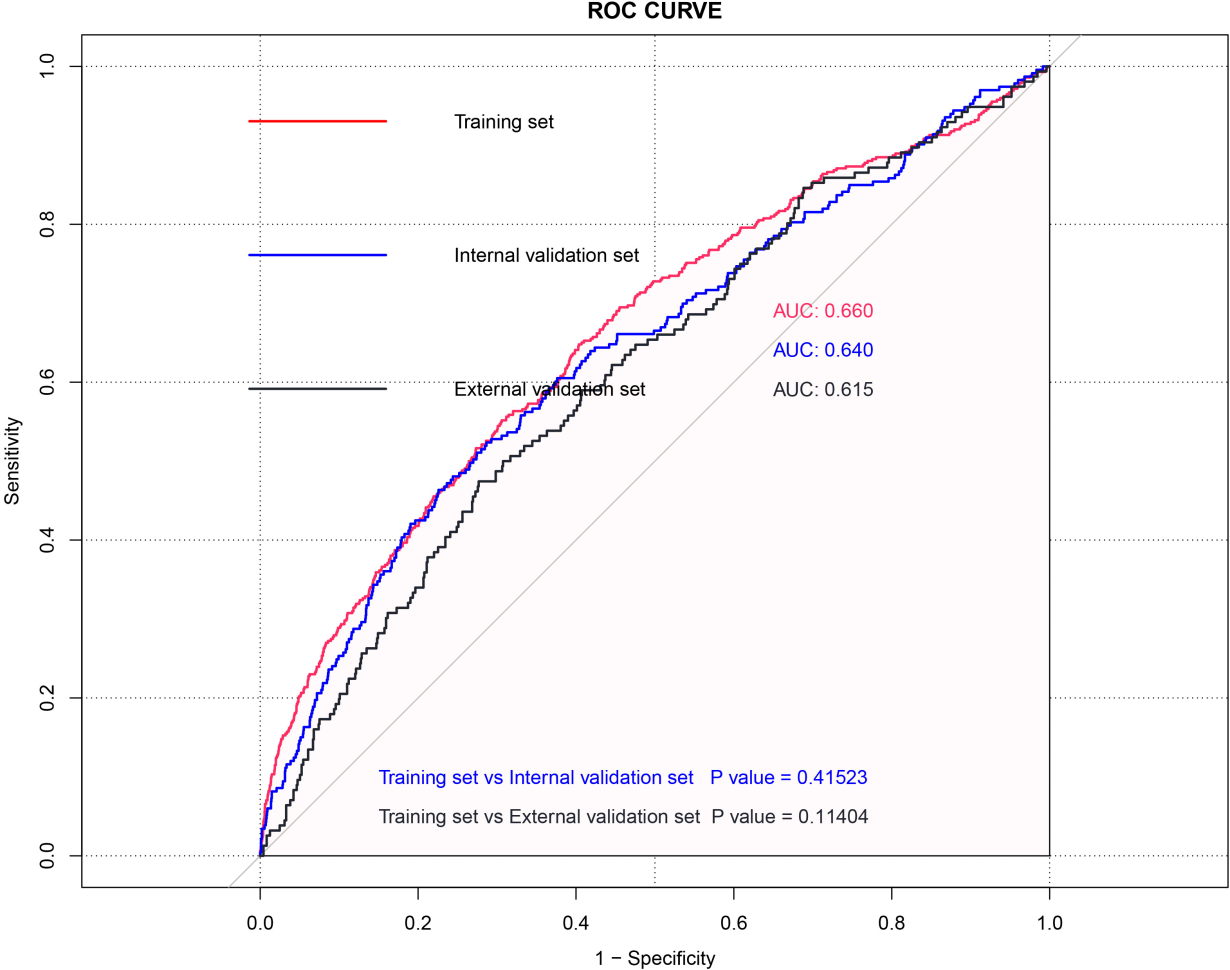
ROC curve of the Training set, Internal validation model and External validation model for total patients.

**Figure 4:**
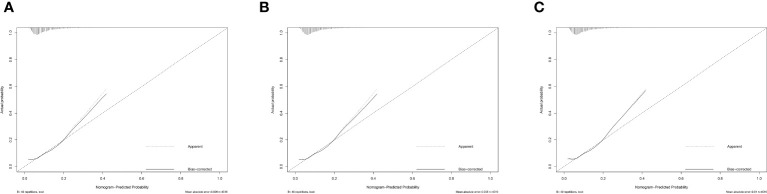
Calibration of the model to predict miscarriage probability in **(A)** Training set, **(B)** Internal validation model and **(C)** External validation model for total patients.

**Figure 5:**
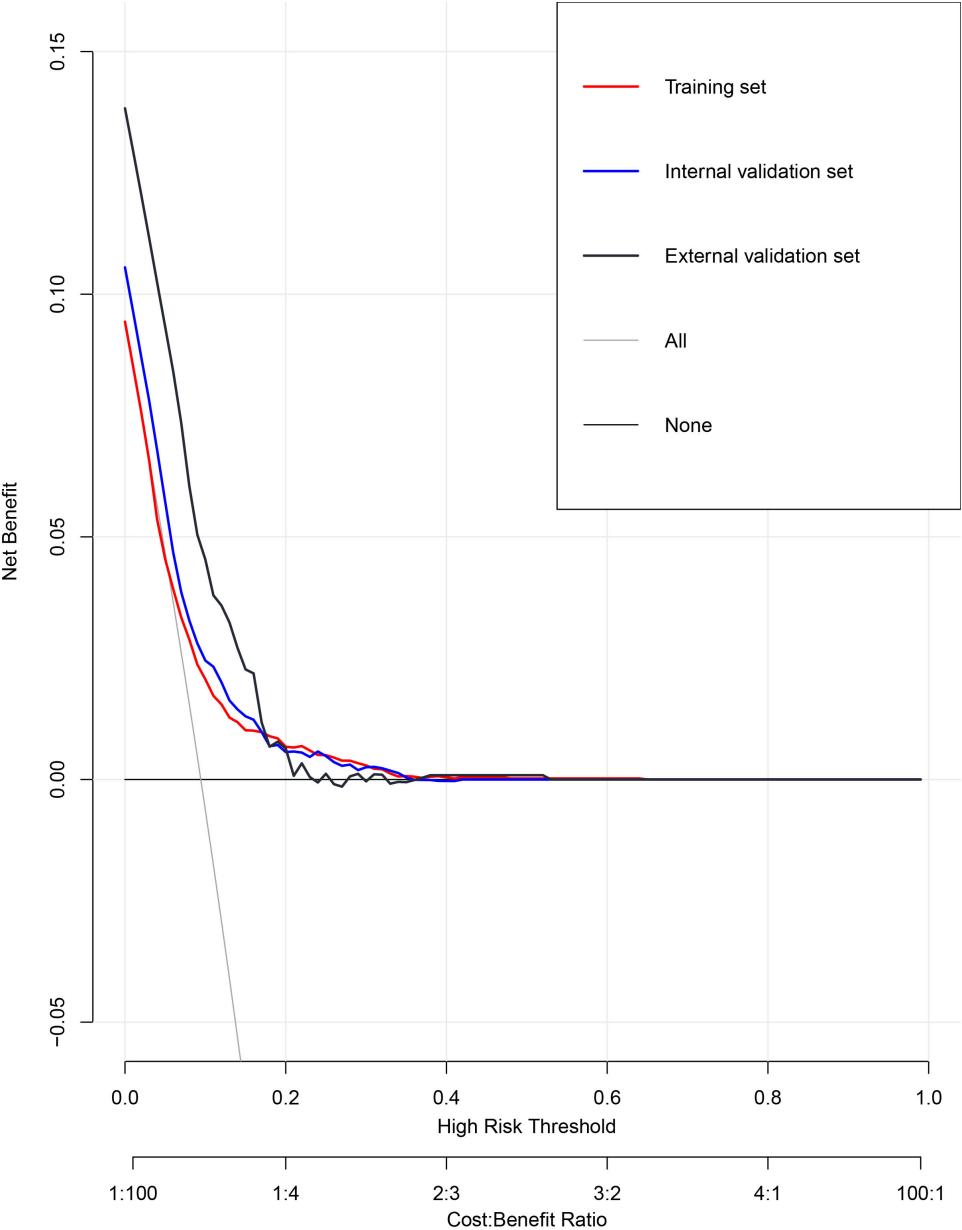
Decision curve analysis of miscarriage nomogram for the total patients.

We then evaluate the performance of this nomogram predict model by AUC, calibration plots, and DCA.The AUC was 0.660 in training sets, 0.640 and 0.615 in internal validation sets and external validation sets, which denoted an acceptable performance (**Figure 3**); Besides, calibration curve also demonstrated good agreement between prediction and observation(**Figure 4**), with P values of unreliability test of 0.845, 0.106, 0.274 for the three datasets. Further, the decision curve (DCA) showed all curves were above the reference line if the threshold probabilities between 0.13 and 0.50 (**Figure 5**). It was proved that the use of this nomogram to predict miscarriage risk was more beneficial than the treat-all or the treat-none schemes.

### Nomogram in patients older than 35 years and less than 35 years

All patients were further segregated into elder (age, ≥35 years, 23.24%) and younger subgroups (age, <35 years, 76.76%). For the elder subgroup, female age (OR=1.26, 95% CI=1.17-1.35, *P*<0.001) and the basal FSH (OR=1.06, 95% CI=1.00-1.11, *P*=0.036) play risk factors on miscarriage, while the number of available embryos (OR=0.92, 95% CI=0.86-0.98, *P*=0.015) can decrease the miscarriage (**Table 2**).As for the younger subgroup, female age (OR=1.05, 95% CI=1.00-1.10, *P*=0.035), BMI (OR=1.05, 95% CI=1.01-1.10, *P*=0.017) and number of spontaneous abortions (OR=1.31, 95% CI=1.02-1.70, *P*=0.038) plays risk roles on miscarriage, while the number of good quality embryos (OR=0.93, 95% CI=0.87-0.99, *P*=0.023) can decrease the miscarriage in multivariate logistic models. Subgroup nomogram were also shown in **Figure 2**.

The AUC of the training dataset, internal validation dataset, and external validation datasets were 0.651, 0.637, and 0.611 for the elder subgroup (**Supplementary Figure 2A**) and 0.653, 0.636, 0,613 for the younger subgroup, respectively (**Supplementary Figure 2B**). The calibration curves also showed that the nomogram probability measurement result and the actual result were consistent (**Supplementary Figures 3A, B**). The DCA in **Supplementary Figures 4A, B** also suggested a better clinical relevance.

## Discussion

This study has developed and validated a nomogram that predicts the probability of early miscarriage in pregnant women undergoing IVF/ICSI treatment. The nomogram takes into account factors such as female age, BMI, history of induced and spontaneous abortions, basal FSH, endometrial thickness on hCG day, and number of good quality embryos. The study group was divided into three subgroups based on age: younger, elder, and general population. The variables incorporated in the nomogram for each subgroup are slightly different, indicating that different factors may have varying roles in predicting the probability of miscarriage in women of different age groups. For instance, the elder subgroup encompasses variables such as basal FSH and the number of available embryos, which may hold greater relevance for women of advanced maternal age characterized by diminished ovarian reserve and lower embryo quality. On the other hand, the subgroup of younger women presents distinct variables that are more relevant to their reproductive health profiles. These variables include female BMI, the number of good quality embryos, and the number of spontaneous abortions. These factors may have a greater impact on the fertility outcomes of younger women.

Our study results showed that the probability of miscarriage increased with advancing maternal age, which is consistent with findings reported by other studies (9, 12, 13). The biological explanation for the higher risk of miscarriage with increasing maternal age is due to abnormal oocyte morphology, declining quantity and quality of oocytes, and an increased risk of chromosomal abnormalities (14). In addition, we conducted subgroup analyses based on maternal age. All patients were divided into younger (age <35 years) and elder subgroups (age ≥35 years). Interestingly, our findings varied slightly between the subgroups. For the elder subgroup, we found that basal FSH levels were a significant factor in predicting the risk of miscarriage, with higher levels of basal FSH indicating a greater likelihood of miscarriage. A study has shown that compared to women with normal basal FSH levels, those with higher basal FSH levels had a higher proportion of aneuploid pregnancies (15). However, the data from Jamie A. M et al. do not provide evidence to support the hypothesis that an elevated basal FSH concentration is linked to an increase in fetal aneuploidy (16). It should be noted that this was a retrospective, single-center study, and the type of stimulation protocol used was not specified. Another study has indicated that the type of stimulation protocol used could potentially affect aneuploidy rates (17). Thus, future studies with larger sample sizes are necessary to address this question. We recommend patients with advanced maternal age maintain confidence and recognize the importance of having a greater number of available embryos. To achieve this, we suggest that older women can improve their pregnancy outcomes by implementing healthy lifestyle practices such as maintaining a healthy weight, abstaining from alcohol and smoking, reducing stress levels, adhering to a healthy diet, ensuring adequate sleep, and engaging in regular exercise.

Regarding the younger subgroup, our study found that BMI is a significant factor associated with miscarriage risk, with an increased probability of early miscarriage observed with higher BMI levels. Some young women with obesity may have misconceptions that they are more likely to conceive as long as they are under the age of 35. However, our study results suggest that young women with obesity should pay more attention to managing their BMI. Sneed ML et al.’s study also revealed that BMI has a significant negative impact on fertility in young patients undergoing IVF, which diminishes as the patient approaches the age of 30, while BMI has minimal impact after the age of 36 (18). Obesity has been linked to a higher incidence of miscarriage (19, 20), This may be due to the high levels of leptin found in obese individuals, which can lead to insulin resistance through altered fatty acid metabolism in skeletal muscle (21), as well as a pro-inflammatory shift in the immune system (22). Insulin resistance is a common symptom observed in obese women (23), and it may be associated with miscarriage through the reduction of insulin-like growth factor-binding protein-1 and uterine αvβ3 integrin (24–26). With the aid of our study results, clinicians can inform patients during consultations that weight loss prior to the IVF/ICSI cycle can decrease the risk of miscarriage.

In addition to BMI, our study identified two other significant factors that impact the miscarriage outcomes of younger subgroup: the number of spontaneous abortions and the number of good quality embryos. As the number of spontaneous abortions increases, the risk of miscarriage also increases, consistent with findings from other published studies (9). The possible reasons may be related to the vascularization of the endometrium. Vascularization of the endometrium is crucial for successful implantation and placentation. Vascular endothelial growth factor (VEGF), an important proangiogenic factor, has been associated with both miscarriage and placental dysfunction disorders when expressed at increased levels in the first-trimester decidua. An increase in angiogenic activity and premature initiation of maternal circulation during early placental development could potentially lead to elevated levels of oxidative stress in subsequent stages (27–29). For women with a history of spontaneous abortions, it is recommended to strengthen the monitoring during pregnancy and provide timely and appropriate drug treatment under the guidance of a physician to decrease the risk of early miscarriage. In addition, from the nomogram model, it is evident that the greater the number of good quality embryos, the lower the risk of early miscarriage. Doctors can advise patients to make lifestyle and dietary adjustments to improve the quality of sperm and eggs and obtain more good quality embryos. Additionally, preimplantation genetic diagnosis (PGD) can be utilized to test the genes of embryos and identify those with specific genetic diseases or defects, which is another way to decrease the risk of early miscarriage.

In addition to the above factors, in clinical practice, the number of induced abortions and medical abortions, and endometrial thickness on the day of hCG are clinical indicators that are considered to potentially contribute to the risk of early miscarriage. Research shows that surgical abortion may cause endometrial injury and lead to complications (30). Induced abortions can potentially damage the endometrium and negatively impact endometrial thickness, which is a crucial factor influencing IVF outcomes (31).Therefore, from a clinical perspective, we have incorporated “endometrium thickness on hCG day” and “history of induced abortions and medical abortions” into our model. The relationship between endometrial thickness and unfavorable outcomes in IVF treatments has been inconsistent across studies. Some articles have reported that a thin endometrium, less than 7 mm, is associated with a higher risk of unfavorable IVF outcomes (32, 33). However, there have been case reports demonstrating successful IVF pregnancies even with minimal endometrial thickness of 4 mm (34), or with increased thickness up to 20 mm (35).Our study results showed that the thinner the endometrium, the higher the risk of miscarriage, which is consistent with findings from a recent multicenter study (36). In our model, although endometrial thickness and history of induced abortions and medical abortions did not reach statistical significance, they still have some line segment length in the nomogram model, which suggests their potential predictive power for the rate of miscarriage. However, it should be noted that the predictive power of these indicators may be weaker than those with statistical significance. Therefore, in clinical practice, we recommend considering these indicators comprehensively rather than solely focusing on those with statistical significance to more accurately assess a patient’s risk of miscarriage. Therefore, for young women, it is particularly important to avoid unnecessary induced abortion, it can disrupt the endometrium, impairing its ability to support and nourish the embryo, leading to thin or poor-quality endometrium and decreased chances of successful pregnancy. If the endometrium is too thin, treatment methods such as estrogen can be used to increase endometrial thickness and decrease the risk of early miscarriage. Additionally, it is important to recognize that miscarriage can have notable psychological effects, such as depression and anxiety (37). Some patients may discontinue further treatment due to the emotional burden of recurrent miscarriage. To alleviate depression or anxiety in these patients, social support and psychological interventions can be employed. Education should be provided first, advising patients to modify their behavior and lifestyle, such as engaging in moderate physical exercise and practicing weight control. Furthermore, if patients are assessed to have a higher risk of early miscarriage, clinicians should inform them to prioritize maternal and fetal health, such as seeking regular prenatal care and receiving sufficient luteal phase support. Additionally, medication dosages should be promptly adjusted based on test results.

Considering the multiple factors that can contribute to early miscarriage, our established nomogram offers a user-friendly and personalized tool for identifying women with a high risk of miscarriage. To our knowledge, no other nomogram prediction model currently exists for predicting risk factors of early miscarriage. The visualized nomogram will improve its clinical usability and enhance its effectiveness in outcome evaluation. Proper nomogram validation is crucial to avoid overfitting and ensure generalizability (38). During model training, we attempted to utilize comprehensive and diverse datasets to ensure the model’s ability to adapt to various data distributions and features. There were no significant differences between the training and internal validation sets, with AUC values of 0.660 and 0.640, respectively, indicating that the model possessed a certain degree of adaptability to different datasets and could exhibit consistent predictive ability across different scenarios. Our external validation population was sourced from the northern region of China, where climate, environment, dietary culture, social culture, and behavioral habits differ significantly from those of the southern region. Upon examination of **Table 1**, it is evident that the external validation group, in comparison to the population used to develop our model, presented with several notable differences. These differences included a higher average age, higher BMI, an increased number of spontaneous abortions, higher Gn dose, longer Gn days, lower number of retrieved oocytes, and lower endometrial thickness on hCG day. Despite these significant North-South disparities between the external validation group and the model development population, our predictive model demonstrated a favorable ability to accurately predict outcomes. Notably, the model achieved an AUC value of 0.615 on the external validation set, indicating its effectiveness in predicting outcomes across diverse datasets and scenarios. These findings underscore the robustness and strong external generalizability of our predictive model. Furthermore, the results indicate that the nomogram exhibits a certain level of discrimination. The calibration curve demonstrates a consistent alignment between the nomogram’s probability measurements and the actual results, attesting to the repeatability and reliability of the developed nomogram. Additionally, Decision Curve Analysis (DCA) was utilized to assess the clinical applicability of our nomogram model, indicating its favorable value in practical clinical settings. In conclusion, through the utilization of both internal and external validation approaches, we have established the robust predictive efficacy of our developed forecasting model. This validation methodology allows for the evaluation of model stability and accuracy across diverse populations in different geographical regions.

Our study possesses several notable strengths that contribute to the advancement of knowledge in the field of assisted reproductive technology (ART). Firstly, it is an observational, multicenter study, which enhances the robustness and generalizability of our findings. By incorporating datasets from both South China as the training and internal validation sets and North China as the external validation set, we have comprehensively examined the applicability of our model across diverse geographical and demographic characteristics. This diversity validation allows us to consider the characteristics, similarities, and differences among populations from different regions, emphasizing the critical implications of applying our research findings to a broader population. Moreover, by validating our model’s performance in both South and North China populations, we have ensured its ability to extrapolate and maintain prediction accuracy across distinct geographical and demographic backgrounds. This validation process strengthens the credibility and applicability of our model, providing further support for its use in clinical practice and decision-making. Secondly, our inclusion of covariates was based on both clinical and statistical significance. We employed Lasso regression and univariate analysis to select covariates, ensuring the accuracy and reliability of our results. This approach strengthens the validity of our model and enhances its robustness, providing a solid foundation for clinical decision-making. Thirdly, the subgroup analysis conducted in this study adds further depth to the research by examining variations in risk factors based on age groups. This information is crucial for clinicians in tailoring interventions and managing patient expectations effectively. Last but not least, our developed model is easily interpretable and presented in a visualized format. This feature facilitates its practical application for infertile couples and clinicians. The model provides realistic and precise information before ART, enabling informed decision-making and the formulation of individualized treatment strategies. By presenting the model’s predictions in a user-friendly manner, we aim to enhance patient satisfaction and improve clinical outcomes. Furthermore, our study emphasizes the benefits of considering population differences. By utilizing data from different regions, we accounted for the diversity among different populations, improving the accuracy and reliability of prediction results. This approach acknowledges that regional variations in lifestyle and dietary patterns can impact reproductive outcomes, ensuring that our model is adaptable and relevant to various populations. We employ internationally recognized diagnostic criteria for infertility, consistent inclusion and exclusion criteria, uniform data collection and analysis methods, and internal and external validation to standardize and assess populations of infertile patients across different regions. These endeavors guarantee the comparability of our research and the reliability of its outcomes, offering a scientific foundation for the application of research results to infertile patients in diverse geographical areas.

Several limitations should be noted. Firstly, although the nomogram has been well-calibrated, significant regional differences between the North and South Chinese populations may have contributed to a slightly lower external validation AUC score. It is important to consider the inclusion of additional prognostic variables, such as behavioral, psychological, and environmental factors, in future studies to further enhance its predictive capabilities. Secondly, the retrospective design of our study may have introduced certain limitations and potential biases. To overcome these limitations, it is advisable that future research endeavors adopt a prospective clinical study design. By incorporating the developed model into such studies, we can thoroughly investigate the effectiveness and suitability of the model in real-time clinical settings, thereby bolstering the strength and reliability of our findings. This approach will yield valuable insights into the performance and practical implementation of the model, ultimately contributing to its wider adoption and improved patient outcomes.

## Conclusion

In summary, our study’s strengths lie in its multicenter design, consideration of clinical and statistical significance, and the practical applicability of the developed model. The user-friendly visualization of results, along with the subgroup analysis, further enhances the value of our research. As we acknowledge the importance of population differences and regional variations, our model remains adaptable and relevant to diverse populations. These findings have practical implications for informed decision-making, individualized treatment strategies, and the overall optimization of ART outcomes. Ongoing research and collaboration are warranted to broaden the scope and impact of predictive models in reproductive medicine, ultimately benefiting a wider population of infertile couples worldwide.

## Data availability statement

The raw data supporting the conclusions of this article will be made available by the authors, without undue reservation.

## Ethics statement

The studies involving humans were approved by the Ethics Committee of The Sun Yat-Sen Memorial Hospital and The Ethics Committee of The First Affiliated Hospital of Xinjiang Medical University. The studies were conducted in accordance with the local legislation and institutional requirements. The patients/participants provided their written informed consent to participate in this study.

## Author contributions

MZ: Conceptualization, Data curation, Formal analysis, Methodology, Project administration, Software, Validation, Visualization, Writing – original draft, Writing – review & editing. XJ: Data curation, Formal analysis, Methodology, Software, Validation, Visualization, Writing – original draft. XH: Data curation, Project administration, Writing – review & editing. YZ: Software, Supervision, Validation, Visualization, Writing – review & editing. HM: Data curation, Validation, Writing – review & editing. HX: Supervision, Writing – review & editing. XL: Conceptualization, Project administration, Supervision, Writing – review & editing, Investigation, Validation, Visualization. QZ: Conceptualization, Investigation, Project administration, Supervision, Validation, Visualization, Writing – review & editing, Resources.
