# Supplementary material for: Development and validation of a visualized prediction model for early miscarriage risk in patients undergoing IVF/ICSI procedures: a real-world multi-center study
Source: Front Endocrinol (Lausanne). 2024 Feb 14;14:1280145. doi: 10.3389/fendo.2023.1280145 (PMC10905617; doi:10.3389/fendo.2023.1280145)
Supplement: Supplementary file 1 [file DataSheet_1.pdf]

Supplementary Material

Supplementary Table 1 Comparison of characteristics and medical history of the study population between training dataset and validation dataset for patients ≥ 35 years.

Variable	Training dataset N1=1045	Internal validation dataset N2=547	External validation dataset N3=311	*P* value
Female age (years)	37.29±2.18	37.22±2.06	37.06±2.02	0.269
Male age (years)	38.98±4.74	38.92±4.29	-	0.806
Female BMI(kg/m^2)	22.06±2.76	22.04±2.71	22.92±2.83[a,b]	<.0001
Duration of infertility (years)	5.28±4.02	5.51±4.01	5.13±3.60	0.349
Type of Infertility				0.030
Primary	298(28.52%)	156(28.519%)	112(36.013%)[a,b]	
Secondary	747(71.48%)	391(71.481%)	199(63.987%)	
Insemination method				0.923
IVF	757(72.44%)	395(72.212%)	-	
ICSI	288(27.56%)	152(27.788%)	-	
Endometriosis				0.628
No	1023(97.89%)	539(98.537%)	306(98.392%)	
Yes	22(2.11%)	8(1.463%)	5(1.608%)	
Tubal factor				<.0001
No	576(55.12%)	312(57.038%)	108(34.727%)	
Yes	469(44.88%)	235(42.962%)	203(65.273%)[a,b]	
Ovulation disorder				0.971
No	1013(96.94%)	531(97.075%)	301(96.785%)	
Yes	32(3.06%)	16(2.925%)	10(3.215%)	
Male factor				0.690
No	907(86.79%)	483(88.3%)	272(87.46%)	
Yes	138(13.21%)	64(11.7%)	39(12.54%)	
Smoking				0.771
No	1038(99.33%)	544(99.452%)	-	
Yes	7(0.67%)	3(0.548%)	-	
Dysmenorrhea				0.034
No	774(74.07%)	379(69.287%)	206(67.763%)	
Yes	271(25.93%)	168(30.713%)[c]	98(32.237%)[a]	
Number of spontaneous abortion	0.25±0.64	0.30±0.68	0.61±0.92[a,b]	<.0001
Number of induced and medical abortion	0.46±0.80	0.45±0.79	-	-
AMH concentration (ng/ml)	8.71±6.94	9.38±7.02	3.08±3.38[a,b]	<.0001
AFC	13.76±7.72	13.71±7.30	9.50±6.93[a,b]	<.0001
Basal FSH (IU/L)	8.34±2.95	8.21±2.89	8.62±3.09	0.146

Basal LH (IU/L)	4.84±2.85	4.94±3.88	4.22±2.42[a,b]	0.003
Basal E2 (ng/ml)	51.21±57.3	59.33± 129	47.16±44.3	0.077
Basal T (ng/ml)	1.36±2.64	1.33±2.11	0.45±0.62a,b	<.0001
Gn dose (IU)	2454.42± 859	2482.05± 892	2995.34± 891[a,b]	<.0001
Gn days	11.23±2.87	11.44±3.22	12.11±2.40[a,b]	<.0001
FSH (IU/L) on hCG day	20.38±8.71	20.68±8.07	-	0.512
E2 (ng/ml) on hCG day	2625.97±1268	2599.45±1259	1747.86±1493[a,b]	<.0001
LH (IU/L) on hCG day	2.27±2.93	2.39±2.18	1.61±1.98[a,b]	<.0001
P (IU/L) on hCG day	1.03±0.54	1.05±0.49	1.79±1.35[a,b]	<.0001
Number of oocytes on hCG day	2.60±1.85	2.57±1.78	-	0.752
Endometrial thickness (mm)	11.79±2.69	11.68±2.57	10.86±2.14[a,b]	<.0001
Retrieved oocytes(n)	10.06±5.03	9.88±4.95	9.14±4.87[a,b]	0.017
2PN	6.36±3.51	6.29±3.52	5.58±3.10[a,b]	0.002
MII oocytes	0.30±0.64	0.25±0.59	4.66±4.68[a,b]	<.0001
Number of available embryos	4.59±2.81	4.79±3.10	4.77±4.48	0.425
Number of good quality embryos	2.14±1.99	2.10±1.98	2.39±2.24	0.115
Number of embryos transferred	1.39±1.21	1.56±1.18[c]	1.91±0.47[a,b]	<.0001
Number of GQB per transferred	1.28±0.80	1.26±0.85	1.35±0.81	0.260
Early Miscarriage				0.188
No	864(82.68%)	444(81.17%)	243(78.14%)	
Yes	181(17.32%)	103(18.83%)	68(21.86%)	

AFC: Antral follicle count; FSH: Follicle-stimulating hormone; LH: Luteinizing hormone;
Basal T: basal testosterone; Gn: gonadotropin; HCG: human chorionic gonadotropins; P: progesterone;
E2: estradiol; MII: metaphase II; 2PN: fertilized oocytes GQB: No. of Good quality blastocyst
a: *P* < 0.05 between External validation dataset and Training dataset.
b: *P* < 0.05 between External validation dataset and Internal validation dataset.
c: *P* < 0.05 between Training dataset and Internal validation dataset.

Supplementary Table 2 Comparison of characteristics and medical history of the study population between training dataset and validation dataset for patients < 35 years.

**Variable**	**Training dataset N1=3471**	**Internal validation dataset N2=1661**	**External validation dataset N3=867**	***P* value**
Female age (years)	29.66±2.88	29.72±2.97	30.14±2.71[a,b]	<0.001
Male age (years)	32.35±4.40	32.28±4.25	-	0.572
Female BMI(kg/m^2)	21.25±2.86	21.15±2.70	22.94±3.42[a,b]	<0.001
Duration of infertility (years)	3.91±2.37	3.92±2.45	3.89±2.47	0.958
Type of Infertility				0.040
Primary	1915(55.17%)	894(53.82%)	512(59.054%)[a,b]	
Secondary	1556(44.83%)	767(46.18%)	355(40.946%)	
Insemination method				0.902
IVF	2480(71.45%)	1184(71.28%)	-	

ICSI	991(28.55%)	477(28.72%)	-	
Endometriosis				0.079
No	3323(95.74%)	1600(96.33%)	844(97.347%)	
Yes	148(4.26%)	61(3.67%)	23(2.653%)[a]	
Tubal factor				<.0001
No	1968(56.7%)	908(54.67%)	261(30.104%)	
Yes	1503(43.3%)	753(45.33%)	606(69.896%)[a,b]	
Ovulation disorder				
No	3295(94.93%)	1572(94.64%)	825(95.156%)	0.841
Yes	176(5.07%)	89(5.36%)	42(4.844%)	
Male factor				0.453
No	2882(83.03%)	1387(83.5%)	707(81.546%)	
Yes	589(16.97%)	274(16.5%)	160(18.454%)	
Smoking				0.391
No	3458(99.63%)	1652(99.46%)	-	
Yes	13(0.37%)	9(0.54%)	-	
Dysmenorrhea				0.038
No	2219(63.93%)	1054(63.46%)	506(59.251%)	
Yes	1252(36.07%)	607(36.54%)	348(40.749%)[a,b]	
Number of spontaneous abortion	0.14±0.42	0.13±0.39	0.36±0.70	
Number of induced and medical abortion	0.25±0.57	0.27±0.60	0.36±0.70	
AMH concentration (ng/ml)	10.56±6.41	10.57±6.44	4.09±3.42[a,b]	<0.001
AFC	18.17±8.67	18.46±8.70	11.84±7.36[a,b]	<0.001
Basal FSH (IU/L)	7.85±2.66	7.83±2.31	7.69±3.52	0.275
Basal LH (IU/L)	5.33±3.58	5.39±3.28	6.61±28.0[a,b]	0.008
Basal E2 (ng/ml)	48.95±73.6	46.60±39.6	55.94±185[a,b]	0.052
Basal T (ng/ml)	1.62±3.39	1.67±3.80	0.77±4.27[a,b]	<0.001
Gn dose (IU)	1998.68± 754	1990.99± 767	2595.07±877[a,b]	<0.001
Gn days	11.52±2.67	11.53±2.63	12.14±2.42[a,b]	<0.001
FSH (IU/L) on hCG day	16.90±6.91	26.89± 371	-	0.113
E2 (ng/ml) on hCG day	2911.17±1257	2953.59±1241	2234.66±1693[a,b]	<0.001
LH (IU/L) on hCG day	2.33±4.01	2.27±2.32	1.39±1.38[a,b]	<0.001
P (IU/L) on hCG day	1.07±0.58	1.06±0.52	2.02±1.43[a,b]	<0.001
Number of oocytes on hCG day	2.96±2.03	2.97±2.00	-	0.871
Endometrial thickness (mm)	11.95±2.59	11.87±2.52	10.87±2.16[a,b]	<0.001
Retrieved oocytes(n)	11.80±5.07	11.96±5.07	11.21±5.30	0.002
2PN	7.44±3.66	7.44±3.62	6.71±3.71[a,b]	<0.001
MII oocytes	0.30±0.67	0.29±0.67	6.38±5.19[a,b]	<0.001
Number of available embryos	5.76±3.30	5.68±3.23	5.61±5.21	0.485
Number of good quality embryos	2.58±2.28	2.54±2.23	2.81±2.60[a,b]	0.017
Number of embryos transferred	1.32±0.92	1.30±0.92	1.83±0.39[a,b]	<0.001
Number of GQB per transferred	1.31±0.73	1.32±0.74	1.37±0.74[a]	0.116
Early Miscarriage				0.003

No	3226(92.94%)	1531(92.17%)	776(89.5%)
Yes	245(7.06%)	130(7.83%)	91(10.5%)

AFC: Antral follicle count; FSH: Follicle-stimulating hormone; LH: Luteinizing hormone;
Basal T: basal testosterone; Gn: gonadotropin; HCG: human chorionic gonadotropins; P: progesterone;
E2: estradiol; MII: metaphase II; 2PN: fertilized oocytes GQB: No. of Good quality blastocyst
a: $P < 0.05$ between External validation dataset and Training dataset.
b: $P < 0.05$ between External validation dataset and Internal validation dataset.
There were no significant differences between Training dataset and Internal validation dataset.

（A）

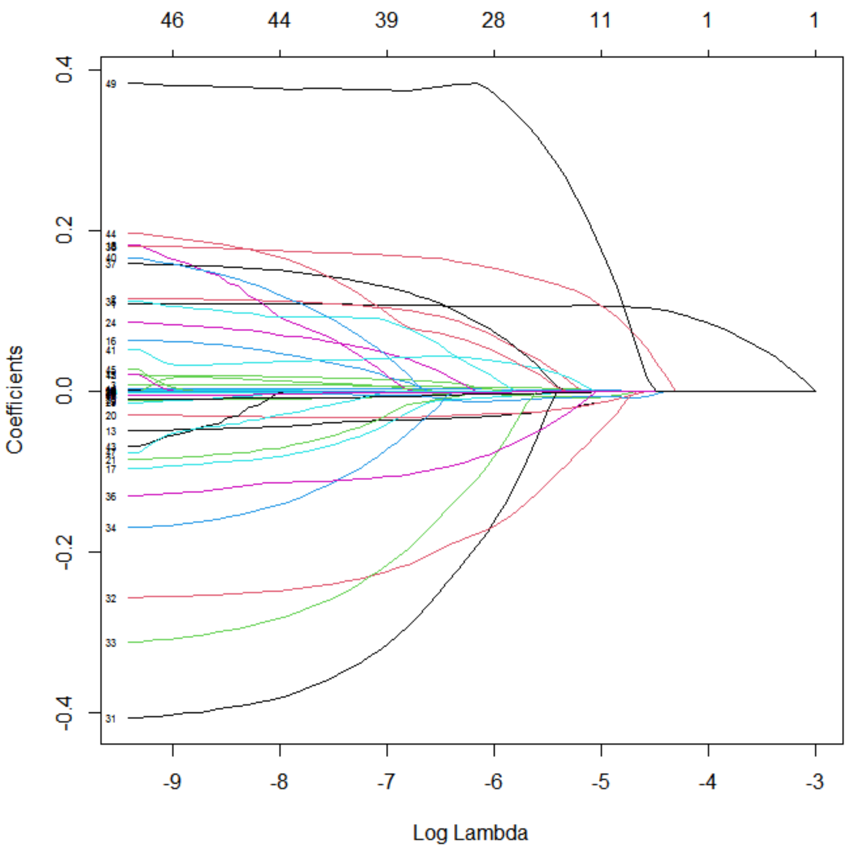

（B）

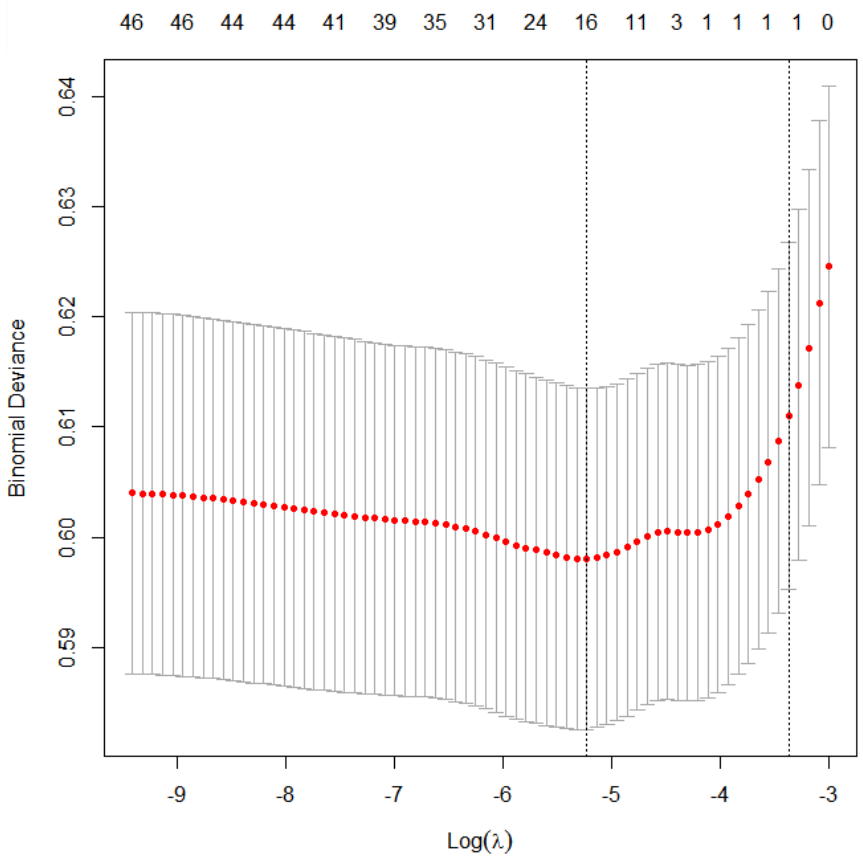

Supplementary Figure 1 (A) LASSO coefficient profiles of variables. Each continuous variable was shown as a colored line; (B) The vertical solid line represents the optimal λ, with which two variables using lambda.1se (left line) or fifteen variables using lambda.min (right line) with non-zero coefficients were screened out.

Supplementary Table 3 Univariable logistic regression models in the train dataset.

Variables	Unmiscarriage N1=4092	Miscarriage N2=424	OR(95%CI)	*P* value
Female age (years)	31.19±4.06	33.65±5.02	1.14(1.12- 1.17)	<.0001
Male age (years)	33.67±5.18	35.94±5.77	1.07(1.06- 1.09)	<.0001
Female BMI (kg/m^2)	21.38±2.83	21.99±3.03	1.07(1.04- 1.11)	<.0001
Duration of infertility (years)	4.20±2.83	4.50±3.42	1.03(1.00- 1.07)	0.042
Type of Infertility				<.0001
Primary	2047(50.02%)	166(39.151%)	1	
Secondary	2045(49.98%)	258(60.849%)	1.56(1.27- 1.91)	
Insemination method				.
IVF	2937(71.77%)	300(70.755%)	1	
ICSI	1155(28.23%)	124(29.245%)	1.05(0.84- 1.31)	
Endometriosis				0.187

No	3933(96.11%)	413(97.406%)	1	
Yes	159(3.89%)	11(2.594%)	0.66(0.35- 1.22)	
Tubal factor				0.030
No	2284(55.82%)	260(61.321%)	1	
Yes	1808(44.18%)	164(38.679%)	0.80(0.65- 0.98)	
Ovulation disorder				0.392
No	3900(95.31%)	408(96.226%)	1	
Yes	192(4.69%)	16(3.774%)	0.80(0.47- 1.34)	
Male factor				0.555
No	3429(83.8%)	360(84.906%)	1	
Yes	663(16.2%)	64(15.094%)	0.92(0.70- 1.21)	
Smoking				0.968
No	4072(99.51%)	424(100%)	1	
Yes	20(0.49%)	0(0%)	0.00(0.00-59E251)	
Male factor				0.915
No	2711(66.25%)	282(66.509%)	1	
Number of spontaneous abortion	0.16±0.46	0.26±0.65	1.40(1.19- 1.65)	<.0001
Number of induced abortion	0.29±0.63	0.38±0.71	1.21(1.06- 1.39)	0.006
AMH concentration (ng/ml)	10.16±6.56	9.88±6.83	0.99(0.98- 1.01)	0.417
AFC	17.28±8.60	15.90±9.22	0.98(0.97- 0.99)	0.002
Basal FSH (IU/L)	7.94±2.70	8.22±3.07	1.03(1.00- 1.07)	0.046
Basal LH (IU/L)	5.24±3.47	4.94±2.98	0.97(0.94- 1.00)	0.083
FSH_LH	12.95±17.9	12.69±11.7	1.00(0.99- 1.01)	0.765
Basal E2 (ng/ml)	49.67±72.4	47.62±43.4	1.00(1.00- 1.00)	0.569
Basal T (ng/ml)	1.57±3.30	1.45±2.56	0.98(0.94- 1.03)	0.469
Gn dose (IU)	2096.64± 801	2176.58± 817	1.00(1.00- 1.00)	0.051
Gn days	11.50±2.73	11.09±2.65	0.94(0.90- 0.98)	0.003
FSH (IU/L) on hCG day	17.63±7.39	18.43±8.52	1.01(1.00- 1.03)	0.037
E2 (ng/ml) on hCG day	2858.89±1259	2712.76±1313	1.00(1.00- 1.00)	0.024
LH (IU/L) on hCG day	2.28±3.73	2.71±4.27	1.02(1.00- 1.04)	0.082
P (IU/L) on hCG day	1.06±0.57	1.02±0.60	0.86(0.71- 1.04)	0.119
No. of oocytes on hCG day	2.89±1.99	2.81±2.04	0.98(0.93- 1.03)	0.446
Endometrial thickness (mm)	11.93±2.60	11.73±2.68	0.97(0.93- 1.01)	0.136
Retrieved oocytes (n)	11.48±5.07	10.60±5.45	0.97(0.95- 0.99)	0.0007
MII oocytes	9.86±4.51	8.93±4.62	0.95(0.93- 0.98)	<.0001
2PN	7.26±3.63	6.56±3.79	0.95(0.92- 0.97)	0.0002
Number of available embryos	5.56±3.24	4.84±3.02	0.93(0.90- 0.96)	<.0001
Number of good quality embryos	2.51±2.23	2.13±2.10	0.92(0.87- 0.96)	0.0007
Number of embryos transferred	1.33±0.99	1.36±1.01	1.03(0.93- 1.14)	0.543
Number of good quality embryos per transferred	1.32±0.74	1.16±0.81	0.76(0.66- 0.86)	<.0001

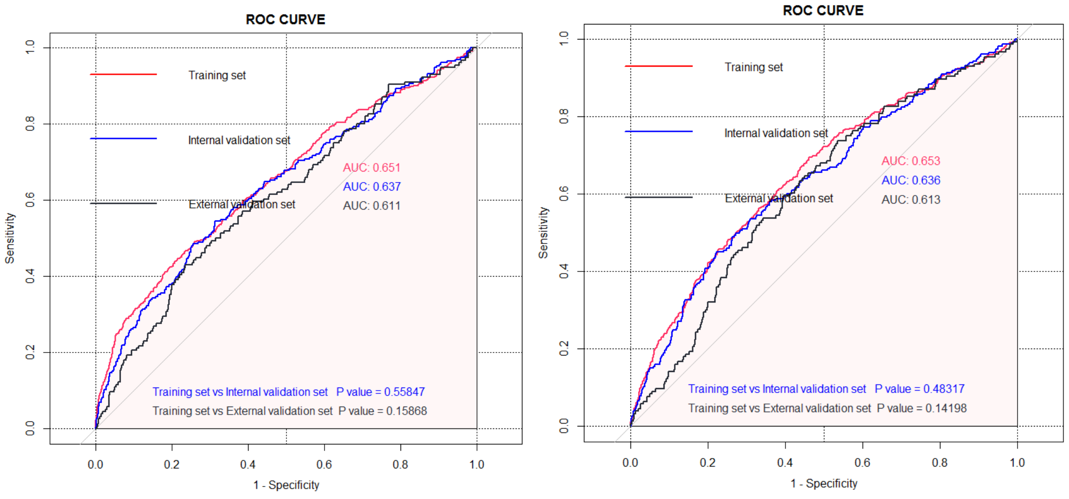

(A) (B)

Supplementary Figure 2 ROC curve for patients ≥35 years (A) and patients <35 years (B).

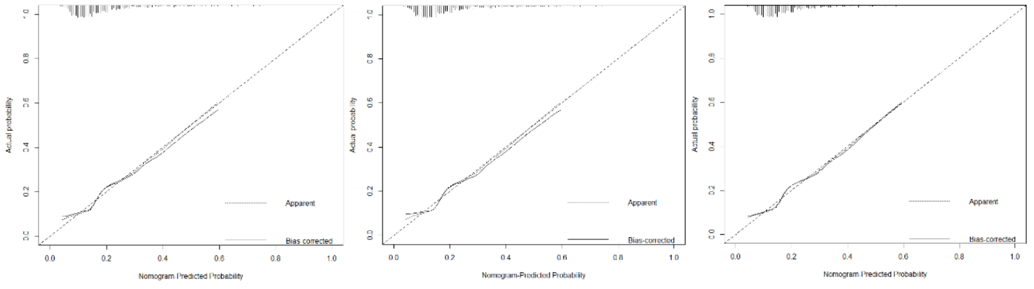

1 2 3

(A)

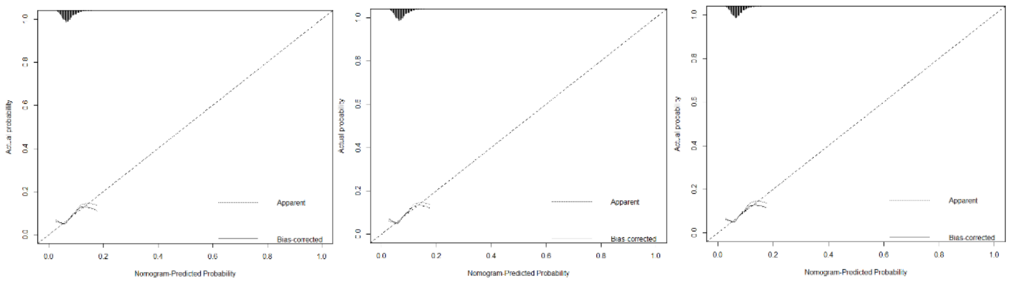

1 2 3

(B)

Supplementary Figure 3 Calibration of the model to predict miscarriage probability in (1) Training set, (2) Internal validation model and (3) External validation model for total patients for patients ≥35 years (A) and patients <35 years (B).

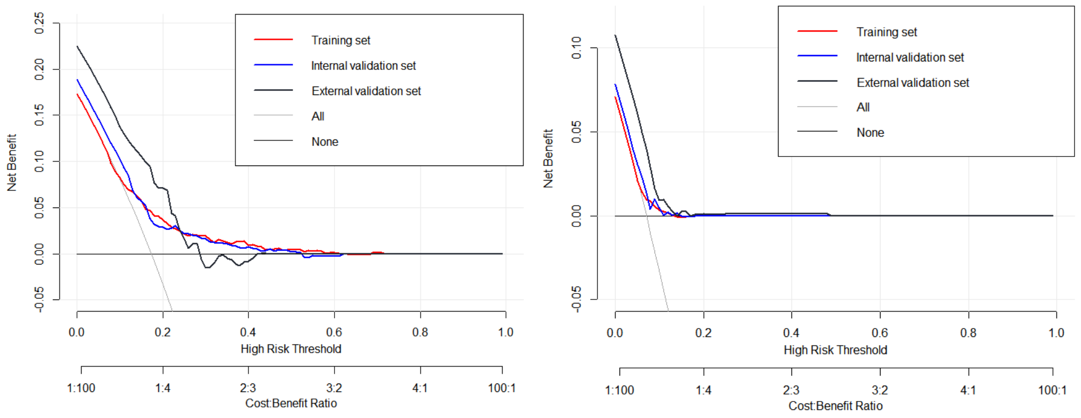

(A) (B)

Supplementary Figure 4 Decision curve analysis of miscarriage nomogram for patients ≥35 years (A) and patients <35 years (B).
